# Explaining Sad People’s Memory Advantage for Faces

**DOI:** 10.3389/fpsyg.2017.00207

**Published:** 2017-02-17

**Authors:** Peter J. Hills, Zoe Marquardt, Isabel Young, Imogen Goodenough

**Affiliations:** ^1^Department of Psychology, Bournemouth UniversityPoole, UK; ^2^Department of Psychology, Anglia Ruskin UniversityCambridge, UK

**Keywords:** mood induction, depression, anxiety, face recognition, eye tracking, face-inversion effect, ownethnicity bias, own-race bias

## Abstract

Sad people recognize faces more accurately than happy people (Hills et al., 2011). We devised four hypotheses for this finding that are tested between in the current study. The four hypotheses are: (1) sad people engage in more expert processing associated with face processing; (2) sad people are motivated to be more accurate than happy people in an attempt to repair their mood; (3) sad people have a defocused attentional strategy that allows more information about a face to be encoded; and (4) sad people scan more of the face than happy people leading to more facial features to be encoded. In Experiment 1, we found that dysphoria (sad mood often associated with depression) was not correlated with the face-inversion effect (a measure of expert processing) nor with response times but was correlated with defocused attention and recognition accuracy. Experiment 2 established that dysphoric participants detected changes made to more facial features than happy participants. In Experiment 3, using eye-tracking we found that sad-induced participants sampled more of the face whilst avoiding the eyes. Experiment 4 showed that sad-induced people demonstrated a smaller own-ethnicity bias. These results indicate that sad people show different attentional allocation to faces than happy and neutral people.

## Introduction

Hills et al. (2011) demonstrated that sad participants were more accurate in a face identity recognition task than happy participants. These authors used a standard old/new recognition paradigm to demonstrate that induced mood affected participants’ ability to accurately recognize facial identity. Furthermore, recognition responses of sad participants were associated with more conscious recollection than that of happy participants. Hills et al. (2011) indicated that the reason for sad participants being more accurate at face processing is that they processed the faces more deeply without explaining what this deep processing is. In this study, we aim to explore why sad people are more accurate at face recognition than happy people by investigating four potential hypotheses: enhanced expert face processing; increased motivation; defocused attention; and more diverse face sampling.

### Enhanced Configural Processing

Face recognition is an expert human ability (Ellis, 1986) that is based on distinct neural underpinnings (Haxby et al., 2002). This neural architecture allows for humans to process faces in an expert manner (e.g., Rossion, 2008). Researchers tend to agree that faces are processed differently to objects (Piepers and Robbins, 2012) and have indicated some form of configural (Tanaka and Farah, 1993) or holistic (Farah et al., 1998) processing as the expert mechanism employed for faces (for a review see Maurer et al., 2002). Indeed, Maurer et al. (2002) distinguish between three forms of configural processing: processing of the first-order relational information (i.e., the basic arrangement of a faces); processing of second-order relational information (i.e., the individual spatial relations between the features in a face (Diamond and Carey, 1986; Carey, 1992); and holistic processing, in which the features of a face and their interrelations are processed as a gestalt whole (e.g., Rossion, 2008)^1^.

In order to assess whether mood affects the amount of holistic processing engaged in, Curby et al. (2009) conducted an experiment in which they measured participants’ performance on the composite face effect (in which two faces are put together to create a new face, Young et al., 1987) following positive and negative mood inductions. The composite-face task is a hallmark of holistic processing (Gauthier and Bukach, 2007; Richler et al., 2008) and identification of the faces that make up the aligned composite is indicative of reduced use of holistic processing (Hole, 1994). Participants induced into a sad mood showed reduced holistic processing, whereas happy participants showed enhanced holistic processing use. Furthermore, Curby et al. (2012) found a significant positive correlation between self-reported positive emotional state and holistic processing use (see also Xie and Zhang, 2015).

While the evidence seems compelling that sad people are less likely to use holistic processing during face recognition, a direct test of all three types of configural processing has not been conducted. The face-inversion effect is often regarded as the gold standard for measuring expertise in face processing (Edmonds and Lewis, 2007). Inversion disrupts all three types of configural processing (first- and second-order relational and holistic information). Upright faces are typically processed in an expert manner, whereas inverted faces have their first-order configuration disrupted and this impacts on face recognition performance.

Given that configural processing has been linked to expert face processing, it is reasonable to predict that there should be a correlation between the amounts of configural processing engaged in and face recognition accuracy. Furthermore, given that sad participants show higher face recognition accuracy than happy participants (Hills et al., 2011), they should engage in more configural processing than happy participants. Given that Curby et al. (2012) found that sad mood is negatively related to holistic processing, we suggest that sad mood causes participants to engage expertise in two of the three components of configural processing (first- or second-order relational processing, rather than holistic processing), and potentially primarily in first-order relational processing given the lack of relationship between second-order relational information and face processing (Hole et al., 2002). In other words, the difference between the results of Curby et al. (2012) and of Hills et al. (2011) is due to the operationalisation of configural coding. In order to measure engagement of all forms of configural processing, the face-inversion effect was chosen for the current study.

### Motivation

A second hypothesis for why sad people are more accurate at face recognition stems from evidence that sad people employ strategies that actively enhance their mood by being more accurate (Clark and Isen, 1982; Erber and Erber, 1994). Sad mood has also been associated with increased elaborative processing and a higher overall recognition of all types of stimuli, compared to individuals in happy mood (e.g., Derryberry and Tucker, 1994; Schwarz and Clore, 1996; Deldin et al., 2001; Deveney and Deldin, 2004). Further work on persuasion suggests that sad people are more likely to process messages deeply in order not to be persuaded by weak arguments (Mackie and Worth, 1991; Bless et al., 1992). In social judgment tasks, sad participants tend to be more accurate than other participants (Lewinsohn et al., 1980). Sad participants tend to be less reliant on using stereotypes (Bodenhausen et al., 1994) and heuristic information (Bless et al., 1996; Hertel et al., 2000) in processing faces (Wyland and Forgas, 2010). This evidence is consistent with the view that sad participants are more motivated to be accurate than happy people. This motivation leads sad participants to process information in a more extensive (Luce et al., 1997) and time-consuming manner. Such motivation may enhance face recognition ability and be revealed by longer time to complete the task (see e.g., Feehan and Enzle, 1991, who have shown that participants who are intrinsically motivated spend longer on tasks than those that are not)^2^.

### Defocused Attention

An alternative way to look at why sad participants might perform differently at a face recognition task, we could look at more general perceptual and attentional differences associated with sad mood. Sad people tend to show enhanced memory for perceptual details, at the expense of the overall picture whereas happy people tend to focus on the ‘gist,’ rather than on details (Gasper and Clore, 2002; Huber et al., 2004). This highlights that sad people do not attend to scenes in the same way as happy people. Focusing on the details can lead sad participants to perform more accurately at various memory tasks.

Attentional focus is also known to be affected by mood. Defocused attention is where attention is broadly focused, unfocused or unselective (e.g., Oatley and Johnson-Laird, 1987) and may lead participants with this attentional set to recall additional contextual information in addition to the target information (Meider and Bröder, 2002; Meiser, 2005). Sad people tend to show defocused attention, in which, during word memory tasks, they are able to recall extra contextual information (such as the side of a screen the word was presented on and the color of the frame surrounding the word; von Hecker and Meiser, 2005). This increased memory for surrounding, extraneous, contextual information may be linked to deeper, more elaborate encoding.

Defocused attention may have consequences for how faces are processed. Typically, when people look at faces, they fixate on the center of the face (Hsiao and Cottrell, 2008). Defocused attention causes extraneous information to the core task to be attended to and potentially stored. In face processing, this might mean that the face identity might be stored with a stronger link to facial expression and other contextual information, such as where the face was encountered, hair styles, jewelry, and other features of a face that are not usually attended to. Defocused attention might lead to elaborative encoding of any extraneous information to the facial identity rather than restrictive focus on the identity. This elaborative encoding might lead to deeper encoding of the expression or jewelry which would typically not be attended to. Moreover, attending to more of the face may mean that more features of the face are actively encoded. Given that only a limited range of features are usually viewed when looking at faces (e.g., Althoff and Cohen, 1999), this may suggest a more detailed representation of the face to be stored in memory. Such additional detail stored may lead to enhanced recognition accuracy. The consequence of defocused attention leading to more facial features being viewed is that the processing of certain types of faces might be altered.

When viewing faces, participants tend to use the most diagnostic features (e.g., Ellis et al., 1975). Hills and Lewis (2006) have indicated that attending to the most diagnostic facial features for the current faces being viewed can lead to increased recognition accuracy. The most diagnostic features for faces varies with facial ethnicity as evidenced from physiognomic variability and eye movement scan patterns. Shepherd and Deregowski (1981) have found greater physiognomic variability for the nose of Black faces than for White faces, whereas the eye (specifically color) varies more for White faces than Black faces. Eye-movement data confirm that participants attend to the most diagnostic features for faces they typically encounter: White participants focus on the eyes when viewing faces (Althoff and Cohen, 1999; Hills et al., 2012). Shorter fixations are made to the remaining internal features with very few fixations to the external features (e.g., Stacey et al., 2005; Hills et al., 2013) leading to the typical triangular scanpath when viewing faces (Yarbus, 1969). East Asian and Black participants tend to focus more on the nose (Blais et al., 2008; Caldara et al., 2010; Hills and Pake, 2013; Miellet et al., 2013). Participants can be made to show enhanced recognition of other-ethnicity faces if their attention is directed to the most diagnostic features for those faces (Hills and Lewis, 2006). Specifically, attending to the nose in Black faces leads to increased recognition of Black faces in both Black and White participants (Hills and Lewis, 2011a). The consequence of this argument is that if sad people are attending to more facial features than happy people as indicated by defocused attention, they may well show a smaller own-ethnicity bias.

### More Diverse Sampling

Depressed individuals avoid eye contact in social situations (Gotlib, 1982). Hinchliffe et al. (1971) suggested that deviation away from the eyes might be extreme, often taking the form of complete refusal to establish or maintain eye contact. Participants induced into a sad mood also avoid eye contact (Natale, 1977). We therefore suggest that eye contact avoidance is a property of mood. Given this, sad people may look to other features in order to recognize faces. Indeed, Hills and Lewis (2011b) have shown that sad people can detect changes made to the head shape and nose of faces better than the eyes, whereas happy people show the opposite pattern. This result indicates that mood affects which features are encoded during face recognition. However, eye-tracking results are necessary to confirm this interpretation.

It is well established that the eyes are the most critical feature of faces for recognition for White participants (Haig, 1986a,b; Gosselin and Schyns, 2001; Schyns et al., 2002; Gold et al., 2004; Vinette et al., 2004) as evidenced by event-related potentials that selectively respond to the eyes (Eimer, 1998) and eye-tracking data showing that the eyes attract more and longer fixations and greater scanning than any other feature (e.g., Walker-Smith et al., 1977; Althoff and Cohen, 1999; Henderson et al., 2001). Ineffective encoding of the eye region holds potential as a link to overall poor recognition of White faces, as it is commonly acknowledged that the eyes have an essential role as the primary focus of an observer’s attention (Hood et al., 2003).

Wu et al. (2012) demonstrated that, during a facial expression recognition task, dysphoric individuals fixated less on the eyes and more on the nose than happier participants. This suggests that sad participants will look at different features to happy participants. In a face identity recognition task, we might initially believe that this should lead to poorer face recognition performance because eye fixation appears to correlate with face recognition accuracy (Hills et al., 2013), it is possible that sad participants actively encode the other features and this may therefore lead to increased face recognition accuracy. We would expect to find eye movement differences when viewing faces between happy and sad participants.

### The Role of Anxiety

While we have presented a number of hypotheses as to why depression, dysphoria, and sadness might affect face recognition, we must not discount the often comorbid (Hirschfeld, 2001) condition of anxiety. While both are associated with emotional disturbances (including eating and sleep disturbances), there are differences between these disorders (such as dysphoria, feelings of worthlessness in depression and feelings of worry and trembling in anxiety). Similar to individuals suffering from depression, clinically anxious individuals show a predisposition for withdrawal from social situations (Öhman, 1986) including an avoidance of making eye contact (Gotlib, 1982).

Individuals exhibiting depression and anxiety disorders have a tendency to scan their environment for signs of impending negative evaluation (Mogg and Bradley, 2002). This could be scanning for a face displaying a critical expression (Lundh and Ost, 1996; Mogg et al., 2000, 2004; Rinck and Becker, 2005; Leyman et al., 2007). Consequently, these individuals develop avoidance strategies in social situations. This could manifest itself through the reduction of conversational eye contact. Eye-contact avoidance can lead to interpersonal dysfunctions (Greist, 1995). In eye movement studies, socially anxious individuals pay less attention to the eye region in comparison to non-socially anxious individuals, spending half as much time exploring the eyes (Horley et al., 2004). Social anxiety is also negatively related to the accuracy with which faces are recognized (Davis et al., 2011). This provides evidence that anxiety may play a role in any relationship between mood and face recognition.

While we might consider that anxiety may play a role in linking depression to face recognition, we must be careful not to conflate depression and sadness, since depression is a clinical disorder involving persistent sad mood, feelings of worthless, and a lack of energy whereas sad mood is a temporary affective state. There is limited evidence that sadness and anxiety are comorbid (e.g., Hesse and Spies, 1994). However, there is evidence that shyness (being nervous in the company of others; a common feature of clinical anxiety) is associated with eye contact avoidance (Iizuka, 1994) similar to that observed for sadness. Those with non-clinical social anxiety also tend to show eye contact avoidance (Farabee et al., 1993). This suggests that some effects of sad mood on face perception relate to a similar mechanism in anxiety, potentially relating to the way in which features are sampled but differences in the depth that they are processed (since sadness is associated with deep processing and anxiety is typically associated with avoidance). In this study, we will address whether the effects of sad mood on face perception are similar to the effects of anxiety (in Experiments 2 and 3).

### The Present Experiment

In light of the background, we aim to understand why sad people show enhanced face recognition performance relative to happy people. We conducted four experiments to test between the various hypotheses presented above and extend the effect to explore its limits. In Experiment 1, we first aim to replicate the findings that mood is associated with face recognition accuracy and test between the first three presented hypotheses: that of enhanced configural processing measured using the face-inversion effect, enhanced motivation, and increased defocused attention. In Experiments 2 and 3, we extend the findings from Experiment 1 and use behavioral and eye-tracking tests to confirm the hypothesis derived from the first experiment. Experiment 4 replicates the findings of Experiment 3 using a different paradigm and in this experiment we test how anger might affect face recognition. In some of the following experiments we used mood induction and in others we measured mood (using measures of depression). Mood induction alters the temporary emotional state of the participant to create happy or sad mood (Hesse and Spies, 1994). Depression is a clinical disorder associated with sad mood, lack of motivation, and feelings of worthlessness. Sad mood associated with depression is often referred to as dysphoria.

Some of the background we have used to justify our hypotheses have been found in depression and others have been found in sad-induced participants. While sadness and depressed mood are distinct constructs with some differential effects on cognition (see e.g., Niedenthal et al., 1999), we have no evidence to suggest that they will affect face recognition differently: nevertheless it is important to explore mood induction, naturally occurring mood (sadness) and dysphoria to fully understand how mood affects cognition. While we used questionnaires measuring symptoms of depression, we were not measuring depression and therefore use the term “dysphoria” from this point forward (similar to Niedenthal et al., 1999; Koster et al., 2005). Therefore, we wanted to test using induction and mood questionnaires to establish if the effects we found are specific to temporarily induced sad mood or dysphoria. In this manuscript, we use the term sad (or sadness) when discussing mood induction or mood induced participants and the term dysphoric (or dysphoria) when discussing naturally occurring moods measured using questionnaires. Without pre-empting the results greatly, had we found differences between results in mood-induced participants and those with naturally occurring moods, we would have explored these.

## Experiment 1

In this study, we aim to understand the memory advantage that sad participants tend to have for face recognition. Firstly, we aim to replicate the finding that mood correlates with face recognition accuracy. We then try to establish why this might be. Several hypotheses have been offered: (1) sad people engage in more configural processing and therefore will show a larger face-inversion effect than happy people; (2) sad people might engage in more effortful processing that happier people and this will be demonstrated through slower reaction times during the recognition task; (3) sad people show defocused attention (von Hecker and Meiser, 2005) and (4) sadness leads to increased attention to more of the face allowing for enhanced featural coding (but not affecting configural processing) and better recognition accuracy. We use a correlational design to address these three hypotheses.

### Method

#### Participants

One-hundred-and-fifty participants aged between 18 and 40 years (mean age = 24.3 years) completed this study. All participants self-reported that they had normal or corrected vision. Participants were recruited via various online platforms. As an incentive to take part, participants were entered into a prize draw to win a £25 gift voucher.

#### Design

We employed a correlational design in which we measured dysphoria using the Hospital Anxiety and Depression Scale (HADS; Zigmond and Snaith, 1983), attentional focus utilizing a source monitoring task of von Hecker and Meiser (2005), and face recognition performance using the Cambridge Face Memory Test (CFMT, Duchaine and Nakayama, 2006). As an index of configural processing, we used the face-inversion effect (Freire et al., 2000): This was calculated as the relative difference in recognition of upright and inverted faces.

#### Materials and Procedure

After providing informed consent, the participants underwent three separate tasks presented sequentially: the dysphoria measure; the attentional focus task, and the face recognition task. All tasks were completed on a computer displayed onto a 15′′ LCD color monitor, using an in-house JavaScript program. Participants made their responses on a standard computer keyboard.

##### Mood Measure

In order to measure dysphoria, we used the HADS (Zigmond and Snaith, 1983). This questionnaire has good internal consistency (Cronbach’s α = 0.78 for depression and 0.81 for anxiety, Bjelland et al., 2002). In this questionnaire, participants read a series of 16 statements that relate to symptoms of anxiety (e.g., “I get a sort of frightened feeling like ‘butterflies’ in the stomach”) and depression (e.g., “I feel as if I am slowed down”). For each statement, participants selected how much the item represents their current mood on a scale of 0–3. The scores from the questions relating to depression symptomology were summed. A score of over 11 represents concern for depression (only four participants scored above the cut-off in the present study, and were already diagnosed with depression). It is widely used in typical populations for its ease and simplicity of use (Whelan-Goodinson et al., 2009).

##### Attentional Focus Test

In order to measure attentional focus, we adapted the source monitoring task of von Hecker and Meiser (2005). This task involved three consecutive phases: learning, break, and a source memory test. In the learning phase, 40 words were presented sequentially on either the left or right of a black vertical line in the middle of the screen in a random order. Each word was presented in Calibri font, size 12, black text on a white background, surrounded in red or green frames (1 pixel wide). Half of the words were presented on each side and half of the words on each side were surrounded by each color frame. Each word was presented for 3 s with a blank inter-stimulus interval of 1 s. Participants were instructed to memorize the word and its position (but the frame color was not mentioned). Upon completion of the learning phase, participants were instructed to take a 30 s break.

Following the break, the source memory test began. Participants viewed 60 words (40 they had seen before and 20 new words) sequentially in a random order in the center of the screen. They were asked, for each word, whether they had seen the word before by clicking the appropriate response on the computer (“Z” for seen before and “M” for not seen before). The word was on screen until the participant responded. If the participant responded with a “yes,” they were then asked what side of the screen the word was on (left or right) and what color the frame was (red or green), responding using the appropriate keys (“L” or “R” and “R” or “G”). The next word was presented immediately after completion of the questions about the previous word.

All 60 words were chosen at random from the word norm database of Paivio et al. (1968) and were nouns of between four and seven letters with between one and four syllables and had a mean concreteness score of between four and seven. The words were drawn at random to be either a target or distractor word and at random to appear on the left or right and with either color frame.

Attentional focus was calculated subtracting the correct recognition of information not to be remembered (i.e., the frame color) from correct answers of the to-be-remembered information (word and position). A higher score indicates more focused attention whereas a lower score indicates defocused attention. This calculation also controls for overall accuracy (von Hecker and Meiser, 2005).

##### Face recognition

The face recognition task consisted of a modified version of the CFMT (Duchaine and Nakayama, 2006), employing the first two stages of the task. After practice trials using cartoon faces, the CFMT contains two separate blocks. In the first block, participants were presented with trials following the same procedure: the same face was presented in the center of the screen three times for 3 s each in three different viewpoints (frontal, 3/4 left, and 3/4 right) sequentially. These images were presented in 7.80 cm wide by 11.35 cm high and 72 dpi resolution. Once the third image was presented, there was a 0.5 s inter-stimulus interval. This was followed by the presentation of three faces side by side. Each face was 5.50 cm wide by 8.00 cm high. One was the target face and the other two were distractors. Therefore, each trial is a 3-alternative forced choice procedure. Participants were requested to select which face they had just seen by keying in the correct button. After this response, a second test screen appeared with the same faces but in a different viewpoint. Again, the participant responded. A third test screen appeared with the same faces but in the final viewpoint. Again, the participant responded. This sequence was repeated for each of six target face identities.

The second block of the CFMT involved participants studying six unfamiliar faces in frontal views for 20 s. These were presented in a 2 × 3 grid. Participants were instructed to memorize the faces. Subsequently, participants’ recognition was tested over the course of 30 further trials, where one of the six target faces was selected among two distractors. The viewpoint of the faces was different to that at learning. Each item was scored as 0 (incorrect) or 1 (correct). This allows for a maximum score of 48 on this test, with chance performance of 16 correct answers. The CFMT was repeated for inverted faces (the order was counterbalanced across participants).

The images of faces used in this study was selected from the database held by Stirling University (PICS, 2014): As in the original CFMT, six identities were used as targets for the upright and a different six identities (counterbalanced across participants) were used as targets in the inverted versions. All faces used within the study were of male individuals aged between 18 and 40 years (mean age 25 years) in order to match the age of the participants (note identical age range to participants) in order to prevent any effects of the own-age bias (Anastasi and Rhodes, 2005; Hills and Willis, 2016). The images were matched for lighting, expression, and pose. All images were presented in grayscale and cropped to exclude hair and clothing as in the original CFMT.

Recognition accuracy for the upright faces gives an overall estimation of face recognition skills. Configural processing was operationalised as the difference between the recognition accuracy of upright and inverted faces divided by the overall accuracy level as this relative measure controls for overall accuracy.

### Results and Discussion

For this and all analyses in this paper, we carefully checked the validity of the data. Any pre-emptive response (defined as a key press less than 500 ms post stimulus-onset) and any protracted response (any response lasting longer than 5000 ms) were removed from all analyses. In no cases did we remove more than 3% of data and this never varied across conditions. While four participants scored above the cut-off on the HADS for depression, we retained their data in the analysis. Removing their scores did not affect the pattern of significance (but did reduce the correlation coefficients).

**FIGURE 1:**
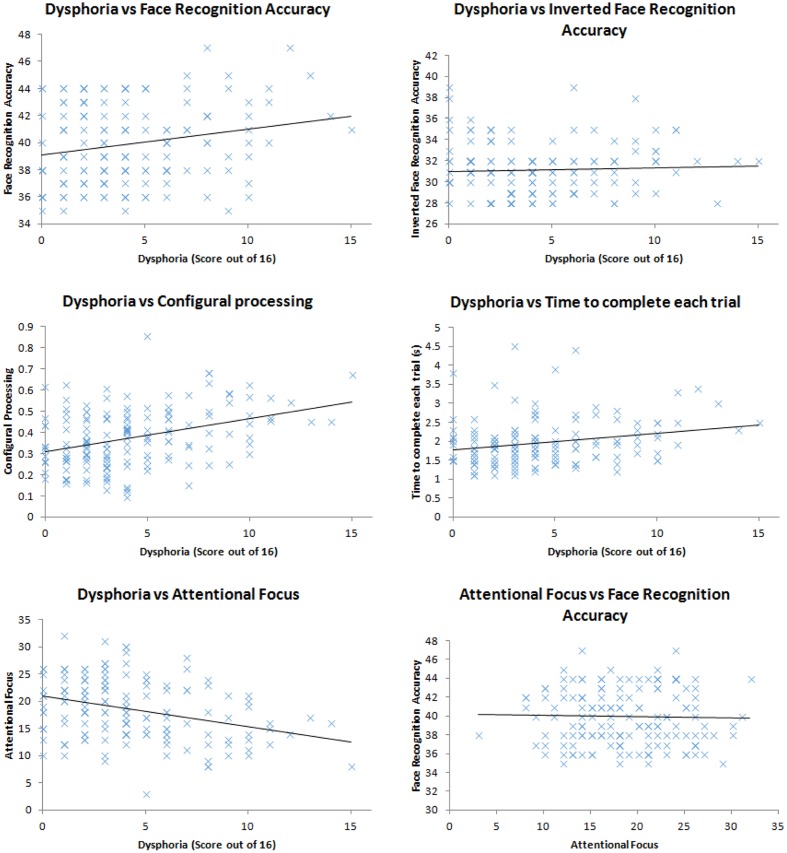
**Scatter plots of the relationship between: dysphoria and face recognition accuracy; dysphoria and inverted face recognition accuracy; dysphoria and configural processing; dysphoria and time to complete each trial; dysphoria and attentional focus; attentional focus and face recognition accuracy**.

We ran correlations between the variables of interest (dysphoria; attentional focus; time to complete the task; face recognition accuracy; configural processing). Firstly, we found that face recognition accuracy correlated with dysphoria, *r*(148) = 0.21, *p* = 0.009, shown in **Figure 1**, replicating the work of Hills et al. (2011). More dysphoric participants were not more accurate at all tasks than happier ones, given the lack of correlation between mood and the recognition accuracy for inverted faces, *r*(148) = 0.05, *p* = 0.561. These correlation coefficients were marginally, but not significantly, different to each other, Fisher’s *r* to *z* = 1.44, *p* = 0.075 indicating that dysphoric participants recognition advantage is primarily for upright faces with which humans tend to employ expert processing mechanisms for. However, dysphoria did not correlate with configural processing, as indexed by the face-inversion effect, *r*(148) = 0.10, *p* = 0.247.

In order to assess whether dysphoria correlated with more engagement with being accurate, we explored the duration with which the participants completed each trial. We assumed that the more motivated participants would engage in more effortful processing to complete the task. This effortful processing, we assumed, would take longer than less effortful processing. We did not find a correlation between time taken to complete the task and dysphoria, *r*(148) = 0.14, *p* = 0.099.

Dysphoria did correlate with attentional focus, *r*(148) = 0.33, *p* < 0.001, replicating the work of von Hecker and Meiser (2005) suggesting that participants who demonstrated higher levels of depression also demonstrated a more defocused attentional style. Defocused attention, however, did not correlate with face recognition accuracy, *r*(148) = 0.03, *p* = 0.757, therefore could not be the cause of sad and dysphoric participants’ impressive face recognition.

Taken together our results indicate that mood (either experimentally induced from Hills et al., 2011, or naturally occurring) is related to face recognition accuracy. However, our three hypothesized mechanisms for this relationship have not been supported by the data: More dysphoric people do not engage in more configural processing than less dysphoric ones; more dysphoric people do not spend more time on the tasks attempting to be accurate; and while more dysphoric people show defocused attention, this attentional set is unrelated to face recognition performance. Therefore, we must look for another mechanism (more diverse sampling of features) for why sad people seem to show superior face recognition ability to happy people. In order to do this, we utilize a method pioneered by Hills and Lewis (2011b).

## Experiment 2

Hills and Lewis (2011b) devised a technique to explore which features are used by participants when they process faces. This technique allows us to identify whether dysphoric participants use a more diverse feature sampling strategy. In their study, participants were required to select which of two faces matched a previously seen face. The distractor faces differed from the target face in terms of changes made to various features. Sad participants were less able to detect changes to the eyes than happy participants, but were more able to detect changes to the outer face shape than happy participants. Potentially, this may indicate that sad participants use different facial features in order to help them recognize faces. In order to assess this, we extended the procedure of Hills and Lewis by exploring participants scoring highly in depressive and anxious traits in order to establish which features they use in face recognition. We tested anxious participants in addition to dysphoric participants’ since anxiety is also associated with avoiding eye contact (Farabee et al., 1993; Horley et al., 2004).

### Method

#### Participants

Sixty (39 female) undergraduate and postgraduate students (aged between 18 and 30 years, mean age = 23.17) at Bournemouth University participated in this experiment voluntarily. Participants self-reported that they had normal or corrected-to-normal vision. Twenty participants were in each group (dysphoric, anxious, or control), see **Table 1** for participant details. We recruited participants until each group had 20 participants in it. Given that participants completed the questionnaires first (see Procedure), if there were sufficient participants in one group, then the Experiment was not continued for further participants in that group.

**Table 1:** Details of the participants for Experiment 1: mean (with standard deviation in parentheses) BDI, STAI, age (years), and gender ratio (female to male).

	Dysphoric Group	Anxious Group	Control Group
BDI Score	22.85 (6.67)	5.55 (3.03)	3.60 (3.80)
STAI Score	88.35 (6.27)	109.30 (10.92)	55.50 (12.21)
Age	22.30 (2.36)	23.50 (3.47)	23.70 (2.39)
Gender (F:M)	15:5	12:8	12:8

#### Design

A 3 × 4 mixed-subjects design was employed with the between-subjects factor of participant group (dysphoric, anxious, or control) and the within-subjects factor of feature change (eyes, mouth, nose, and outer head shape). Feature changes were distinguished by either enlargement or reduction of the feature or replacement of the feature entirely. Counterbalancing was employed such that each target face had an equal number of appearances for each experimental condition. The type of feature change and type of facial expression were also counterbalanced and appeared an equal number of times throughout the experiment.

#### Materials

**FIGURE 2:**
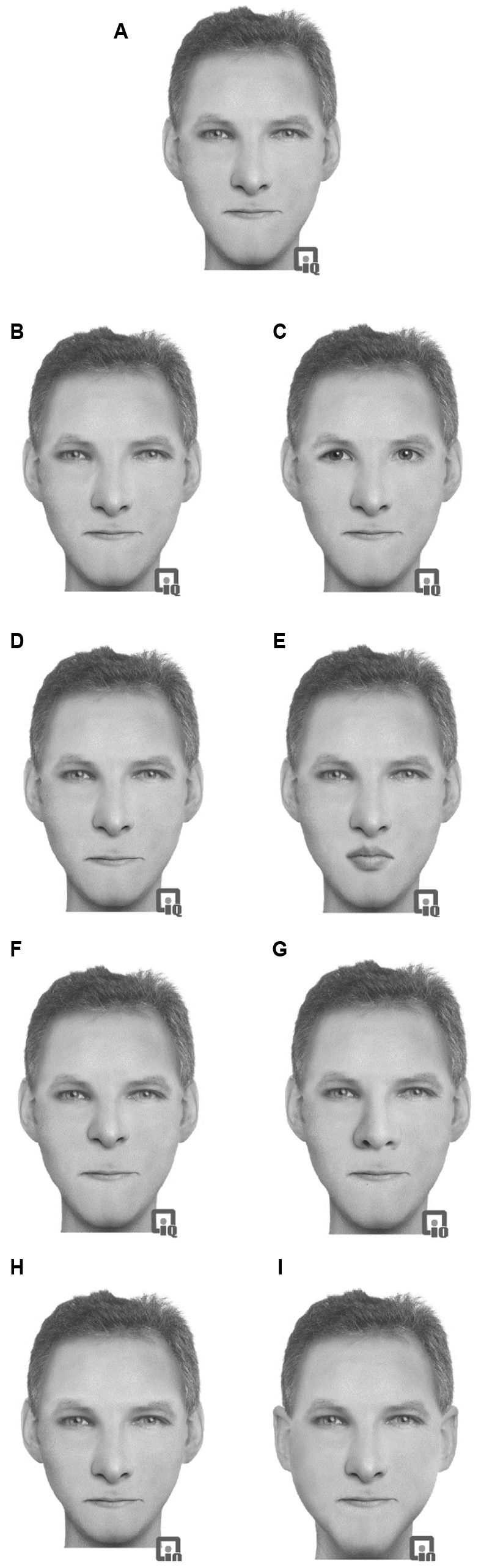
**Example of the stimuli used in Experiment 2 with an original prototype (A)** face with modifications: **(B)** eyes changed, **(C)** eyes replaced, **(D)** mouth changed, **(E)** mouth replaced, **(F)** nose changed, **(G)** nose replaced, **(H)** outer head changed, **(I)** outer head replaced.

Sixteen prototype faces were constructed using the face-reconstruction software Faces 3.0 (InterQuest^TM^). This computer-based software allows facial construction from a set of saved features (e.g., head shape, chin shape, eyes, eyebrows, nose, and mouth). Each feature can be adjusted and repositioned, shrunk, or enlarged in relation to the other features to create a wide variety of faces. Sixteen (eight female) ethnically White prototype faces were constructed. This construction allows for realistic faces to be constructed and for features to be moved without any disruption to the facial image (through stretching or morphing) and has been used in previous studies (e.g., Hills and Lewis, 2006, 2011b). The images were presented in grayscale on a 13.3′′ screen. While these images were not photographs of faces, they maintain the important texture, surface reflectance, and shape information of the faces which are critical to expert processing of faces (Russell et al., 2007). Full color images only enhance face recognition if shape and surface reflectance information is unavailable (Yip and Sinha, 2001). In this case, we anticipate the images to be of similar quality to photographs and therefore will produce face-like responses. Each of the 16 prototype faces was constructed from a unique set of facial features that were not shared by any of the other prototype faces. Eight modified versions of each prototype face were constructed by making changes to the eyes, mouth, nose, or outer face (see **Figure 2**). Note that the hair style was not changed when the outer face was changed. The outer face changed the ears and the chin shape. Each face was given a common one-syllable name dependent on the sex.

To examine anxiety symptoms within participants, the study utilized the State-Trait Anxiety Inventory (Spielberger, 1983). The STAI is a 40-item instrument of self-report scales measuring transient and enduring levels of anxiety. STAI State question items pertain to examine how the respondent is currently feeling (e.g., “I feel at ease”), with responses ranging on 5-point scales from ‘not at all’ to ‘very much so’. STAI Trait items assess how respondents generally feel (e.g., “I am a steady person”), with responses ranging from ‘almost never’ to ‘almost always.’ This scale is well established as a diagnostic tool in research, with strong psychometric support and adequate internal consistency (Stanley et al., 2001; Spielberger and Reheiser, 2004). A cut-off of around 88–102 indicates anxious individuals (Kaneda and Fujii, 2000; Stanley et al., 2001; Stark et al., 2002).

The Beck Depression Inventory (BDI: Beck et al., 1961; Beck and Steer, 1987) was used to measure depressive symptoms. It consisted of 21 multiple-choice self-report questions. The BDI has internal consistency at least α = 0.75 (Richter et al., 1998). We used a different measure of mood in Experiment 2 to Experiment 1 since there is often a concern in research that correlations and experimental effects may be a result of the specific measures used. By using a different measure, it provides more confidence in the generalisability of these results. Severity of symptoms ranges from minimal to severe responses (e.g., “I do not feel sad”; “I am so sad or unhappy that I can’t stand it”). Individuals respond to the statement that best fits their emotional state during the past 2 weeks. This instrument is one of the most widely used psychometric tests for measuring depression severity. Furthermore, well-grounded research on BDI has inferred that 0–13 on the scale is associated with mild depression (Smarr and Keefer, 2011), hence the cut-off point within this study was 13.

#### Procedure

After providing informed consent, participants completed the STAI (Spielberger, 1983) and the BDI (Beck and Steer, 1987) sequentially. Participants were next allocated to one of three experimental groups dependent on their overall scores for the two questionnaires. No participant scored high in both the STAI and the BDI, therefore we were able to allocate participants to either a depressed, anxious, or control group. Following this, participants underwent the face processing task involving a learning phase followed by a test phase.

Participants began the learning task. Sixteen prototype target faces were sequentially presented on screen in a randomized order, with a 150 ms noise mask displayed between each face. Faces were presented in the dimensions 194 × 238 mm during this stage. Participants were instructed to learn the 16 face-name pairings. Progression onto the next screen was response terminated. Following this, the same 16 faces appeared sequentially for 3000 ms, and a one-syllable name appeared beneath each face. Between each of the 16 consecutive trials, a 150 ms random mask noise appeared. Participants were represented with the 16 prototype faces without the names and were asked to identify through a two-alternative forced-choice test which face correctly matched the corresponding name. Faces were presented in the dimensions 230 × 297.5 mm during this stage. The two names from which the participants had to choose from were names which they had previously learnt. Selection of the two names was achieved by clicking the corresponding keys, either ‘1’ (name on the left) or ‘2’ (name on the right), which was response terminated. Feedback was provided to participants. This task ensured that participants retained a perceptual memory of each face. Participants reached a mean accuracy level of 94.04% (anxious participants), 94.06% (control participants), and 88.75% (dysphoric participants) in this task, which indicated they had learnt to match all but one name, on average. There were no differences across participant groups on the face-name learning, *F*(2,57) = 1.11, *MSE* = 0.02, *p* = 0.337, $\eta_{p}^{2}$ = 0.04.

After the learning phase, participants were presented with the test phase: a two-alternative forced-choice test in which two faces were presented simultaneously, side by side. The face images were presented with a dimension of 92 × 119 mm in this phase (the use of different sized images reduces the impact of low-level picture matching). Each test trial involved only one type of facial alternation alongside one of the original prototype faces. Participants were then instructed to identify the face in which they had originally learnt via the question “Which face is [Bill]?”. Participants communicated their answer through pressing the appropriate key: ‘1’, indicating the face on the left, or ‘2’, indicating the face on the right. Each of the 16 trials were response terminated. Between each trial was an inter-stimulus interval random noise mask lasting 150 ms. Each of the 16 trials were presented in a randomized order, where the correct answer was randomized to either the left or right side of the screen, with each participant only viewing each of the 16 prototype faces once.

### Results and Discussion

**FIGURE 3:**
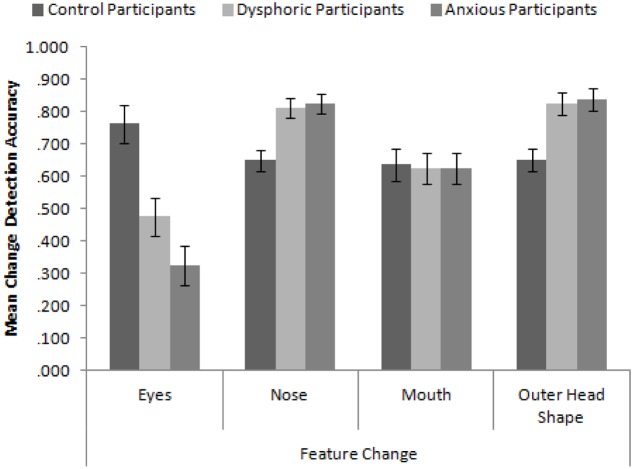
**Mean feature change detection accuracy for control, depressed, and anxious participants split by type of feature**. Error bars show standard error.

In order to replicate the findings of Hills and Lewis (2011b), we calculated proportion accuracy of identifying the correct face across the four conditions of feature change. We first ran a 3 × 4 mixed-subjects ANOVA with the factors participant group (high in anxiety symptoms, high in dysphoria, and control) and type of feature change. The mean accuracy to detect these changes is presented in **Figure 3**. Consistent with Hills and Lewis (2011b), the interaction between participant mood and feature was significant, *F*(6,171) = 10.58, *MSE* = 0.043, *p* < 0.001, $\eta_{p}^{2}$ = 0.27. In order to explore this interaction, we ran a series of independent-samples *t*-tests comparing the accuracy of feature change detection across the different participant groups. We employed the Bonferroni-correction for multiple comparisons (α = 0.004). These revealed that control participants detected changes to the eyes more accurately than dysphoric participants, *t*(38) = 3.28, *p* = 0.002, and anxious participants, *t*(38) = 5.32, *p* < 0.001. Dysphoric and anxious participants detected changes to the nose, *t*(38) = 3.32, *p* = 0.002 and *t*(38) = 3.52, *p* = 0.001 respectively, and outer head shape, *t*(38) = 3.31, *p* = 0.002 and *t*(38) = 3.51, *p* = 0.001 respectively, more accurately than control participants. There were no differences in accuracy between sad and anxious participants (all *p*s > 0.093).

These results replicate and extend the findings of Hills and Lewis (2011b) in several important ways. Firstly, we have established that control participants appear to be attending to the eyes more than all other internal and external features (Hills and Lewis, did not test the mouth). Secondly, sad (in this case, dysphoric) and anxious participants are better at discriminating changes made to the nose and head shape than the mouth and eyes. Indeed, they are significantly worse at detecting changes to the eyes than control participants but significantly greater at detecting changes made to the nose and head shape than control participants. These results seem to suggest that sad and anxious participants are actively avoiding the eyes and are showing a more defocused attentional style when viewing faces. However, they do not appear to be using the mouth any more than control participants suggesting that the mouth may not be a useful feature for face recognition.

Finally, we have extended the results of Hills and Lewis (2011b) into naturally occurring moods rather than experimentally induced ones. This is an important addition as it demonstrates that the results of Hills and Lewis (2011b) were not due to demand characteristics. It also suggests that there are not qualitative differences between artificially induced moods and naturally occurring ones in their effect on face matching.

In terms of our overall question, these results seem to suggest that sad and dysphoric people tend to employ a strategy that encourages encoding of more facial features than would be typical. Precisely, they are using external features and the nose more than control participants do. Typically, viewing the external features does not lead to increased recognition accuracy (e.g., Hills et al., 2013). Viewing the nose leads to increased face recognition accuracy in other-race faces (Hills and Pake, 2013), but should not have such a large effect in the recognition of own-race faces given the importance of the eyes in face processing (e.g., Eimer, 1998). Focusing on the nose has been linked to enhanced holistic processing (Schwarzer et al., 2005; Kelly et al., 2010) which may lead to increased recognition accuracy (Van Belle et al., 2010). Potentially, sad participants are actively processing all features in order to recognize faces accurately. In order to confirm this prediction and see if it actually does lead to greater recognition accuracy, we conducted a third experiment employing eye-tracking.

## Experiment 3

Our results indicate that psychometrically measured dysphoric participants are more accurate at face recognition than neutral participants and this seems to be related to defocused attention and eye avoidance because dysphoric participants can discriminate changes made to parts of faces (the nose and head shape) that control participants are unable to detect. This may be the result of an atypical scan pattern relative to those found in control participants when looking at faces. In order to assess this directly, we employed a recognition paradigm in which participants’ eye movements were recorded as they processed faces. Eye-tracking can be used to measure the focus of attention (Phillips and David, 1994; Leonards and Scott-Samuel, 2005; Gilchrist and Proske, 2006; Morand et al., 2010), given that the function of eye-movements is to move parts of an image to the high-resolution fovea allowing for critical information to be focused on (Desimone and Duncan, 1995; Egeth and Yantis, 1997), though there is not a one-to-one relationship between fixation point and information encoding.

In contrast to the previous experiments, we used a mood-induction procedure. We had wanted to show the effects of mood on face perception that have been found previously (Hills and Lewis, 2011b; Hills et al., 2011) occur in naturally occurring moods rather than only in induced moods. Experiments 1 and 2 have shown this. Now we want to further explore the relationship with mood and face perception and the mood induction technique is a more flexible technique: In this experiment, we also wanted to compare sad mood with happy mood as this is an element missing from psychometrically measuring dysphoria (as in Experiments 1 and 2). Previous research indicates that the effects of mood on how people view faces occurs irrespective of whether the mood is naturally occurring or caused by induction (see Natale, 1977; Gotlib, 1982). We, therefore, chose to use a mood-induction procedure that would ensure we would get a sufficient number of happy and sad participants to compare the effects of mood on eye movements.

In this Experiment, we also wanted to further explore how anxiety affects how faces are viewed. We demonstrated in Experiment 2 that anxiety produced similar findings for feature change detection as sad mood. There is significant comorbidity between anxiety and depression (Hirschfeld, 2001) but not significant comorbidity between sad mood and anxiety, therefore, we used this experiment to further show that the results found in sadness are not unique to sadness but rather due to a common issue with anxiety. We predict that sad and anxious participants should avoid looking at the eyes during the recognition task in favor of looking at other features.

In this Experiment, we further aimed to assess whether there was any impact of facial expression on where participants would look. Therefore, we used four different facial expressions: happy, sad, neutral, and fearful. We chose these expressions to establish if any mood-congruent recognition biases occur. We also used upright and inverted faces in order to see whether mood affected the amount of configural coding used (although we anticipate a null finding here, due to the results presented in Experiment 1).

### Method

#### Participants

Sixty (40 female) participants from Anglia Ruskin University were recruited for this study. All participants self-reported that they were ethnically White and had normal or corrected vision.

#### Design

We employed a 4 × 4 × 2 mixed-subjects design with the between-subjects factor of participant mood (happy, sad, anxious, and neutral) and the within-subjects factors of facial expression (happy, sad, fearful, and neutral) and facial orientation (upright and inverted). The dependent variables in this experiment were face recognition accuracy measured using the Signal Detection Theory measure *d*’ and the total fixation duration to each feature. The presentation order of faces in the learning and test phases was fully randomized. Faces were counterbalanced across participants such that they appeared as a target and as a distractor an equal number of times. Moreover, faces appeared with each expression an equal number of times. This study received full ethical approval from Anglia Ruskin University’s Faculty of Science and Technology Research Ethics Panel.

#### Materials

We used the NimStim face (Tottenham et al., 2002); Karolinska Directed Emotional Faces (Lundqvist et al., 1998); and Psychological Image Collection at Stirling (PICS) databases. These databases contains a series of faces with their expressions validated by independent observers and are widely used in face recognition research. These images were cropped to have the same background and all clothes masked. We employed eight images of each face: two of each expression – one was used for learning and the other for test. This helps to minimize pictorial recognition. These were counterbalanced across participants. Two-hundred and forty individual face identities were used. The images were constrained to 506 pixels wide by 764 pixels high and were presented in grayscale with a high resolution (106 dpi).

All stimuli were displayed on a white background in the center of a 17′′ (1280 × 1024 pixels) LCD color monitor. The stimuli were presented and recognition responses were recorded using E-Prime Profession Version 2 and eye movements were recorded using a Tobii 1750 eye-tracker (Falls Church, VA, USA), with embedded infrared cameras with a sampling rate of 50 Hz. The eye-tracker emits near infrared light, which reflects off a person’s eyes, which is then detected by the eye-tracker’s camera. A fixation was defined as the eyes remaining in the same 30 pixel area for at least 100 ms (see Goldinger et al., 2009). If the eyes left the region, but returned within 100 ms, it was considered to be the same gaze. These settings were based on the defaults for the Tobii eye-tracker. Participants’ heads were restrained using a standard chinrest 65 cm from the monitor.

We used the Visual Analog Scale (VAS: Aiken, 1974) to measure mood. It consists of a 100 mm line with the anchor points “extremely positive mood (happy)” at one end and “extremely negative mood (unhappy)” at the other end. Participants mark down on the line the point that best reflects their mood. Mood is therefore operationalised as the point along the line the participant marked measured in millimeters. The VAS is a reliable measure of mood (Ahles et al., 1984).

To induce mood, an autobiographical memory task, based on Hesse and Spies (1994) was used. Participants were told to:

“Write down [the happiest/saddest/most anxiety inducing moment of your life/your journey to University today]. You have 5 min to complete this task. Please be as accurate and emotive as possible. Be assured that your information is completely anonymous.”

Note that the “neutral” induction involved writing about the participants’ journey to university, therefore meant that the neutral participants were treated in as similar manner as the other participants as possible. Participants wrote their memories down on a plain piece of paper with no identifying information. They had 5 min to complete this task. These memories were destroyed at the end of the experiment.

#### Procedure

After providing informed consent, participants were asked to complete the VAS to estimate their current mood. Participants’ mood was then induced using the autobiographical memory task.

Once the participants completed the mood induction, they were positioned in front of the computer monitor with the keyboard directly in front of them and their head placed comfortably on a chin rest to keep head movement to a minimum. Their hand was placed over the relevant keys on the keyboard in order to minimize movements. Participants’ eyes were then calibrated to the eye-tracker using the in-built calibration system. This required the participants to track a moving blue circle around a white background to nine pseudo-random locations on the screen. From this point, there were three consecutive phases: the learning phase, distraction, and the test phase.

In the learning phase, participants were told that they would see a series of faces and would have to rate each face according to the question “how easy would this face be to spot in a crowd?” (a measure of distinctiveness: Light et al., 1979). To make this rating, participants used a one to seven scale, with the anchor points “easy to spot in a crowd” (distinctive face) and “difficult to spot in a crowd” (typical face). Participants were presented with 120 different face identities sequentially in a random order (faces were split equally among the eight conditions, therefore there were 15 faces of each type presented). Participants were instructed to rate the face whilst it was on screen using the numerical keypad on the computer keyboard. Faces remained on screen for 3 s. Between each face, a random noise mask was presented for 150 ms.

Immediately following this task, the distractor phase began. This involved participants completing the VAS for their present mood (i.e., their mood following the mood induction) and then provide some demographic information (age, gender, place of birth, and place of residence). Participants’ eyes were then recalibrated to the eye-tracker using the same procedure as described above. This phase took roughly 60 s to complete.

Participants then were given the instructions of the standard old/new recognition test phase: to state, for each face, whether they had seen it before by pressing the appropriate key on the keyboard. The keys were “z” for seen before and “m” for not seen before. Participants were presented with all 240 faces (120 targets and 120 distractors in the same proportion of expression and orientation as in the learning phase) sequentially and in a random order. Each trial was response terminated. Between each presentation a mask of random noise was presented lasting 150 ms.

After the final face was presented, all participants were instructed to complete the happy mood induction in accordance with ethical guidelines. Participants were thanked and debriefed.

### Results and Discussion

In order to check that the mood induction procedure worked, we calculated the difference between the participants’ current mood (collected halfway through the experiment) and their average mood (collected before the mood induction). A greater difference in this indicated a more successful mood induction^3^. Mood was significantly affected by induction, *F*(3,56) = 37.23, *MSE* = 29.75, *p* < 0.001. Bonferroni-corrected *post hoc* tests demonstrated that happy induced participants were happier than all other participants (all mean differences >9.30, *p*s < 0.001) and that sad and anxious induced participants were sadder than neutral and happy participants (all mean differences >6.02, *p*s < 0.023). Numerically, sad induced participants were sadder than anxious participants (mean difference = 4.57) but not significantly (*p* = 0.153). This demonstrates that our mood induction was effective.

We present the behavioral and eye movement data separately for clarity. For all comparisons throughout these results, when Mauchley’s test of sphericity was significant, the Huynh and Feldt (1976) correction was applied. This was chosen since the sphericity estimates were typically above 0.75 (Girden, 1992). Here, we report the corrected significance levels but the uncorrected degrees of freedom.

#### Eye-Tracking

**FIGURE 4:**
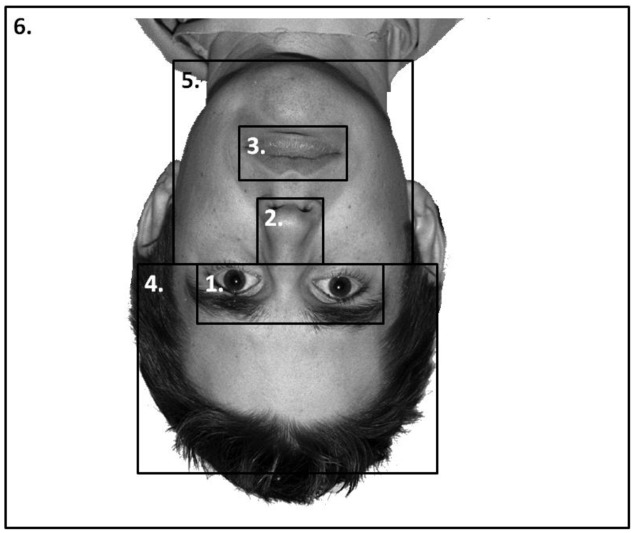
**An example stimulus with the AOIs mapped onto it: (1) Eyes; (2) nose; (3) mouth; (4) forehead; (5) chin and cheeks; and (6) the rest of the screen**. AOIs were not visible to the participants.

Six areas of interest (AOIs) were mapped out on to each individual stimulus independently in a similar manner as Goldinger et al. (2009, see **Figure 4**). These mapped out areas were not visible to participants. The areas were based on theoretically important regions of the face. We analyzed the duration of fixation in each AOI until the participants’ responded in both the learning and the recognition phase of the experiment. Due to the AOIs occupying vastly different amounts of the screen, we conducted an analysis on area-normalized AOIs (calculated by dividing the proportion of fixations or durations by the proportion of the screen the AOI occupied, see Fletcher-Watson et al., 2008; Bindemann et al., 2009). A value of 1 indicates the AOI was scanned at chance levels; a value above 1 indicates the AOI was scanned more frequently than chance levels. The pattern of results from a non-normalized analysis was identical to that presented here.

**FIGURE 5:**
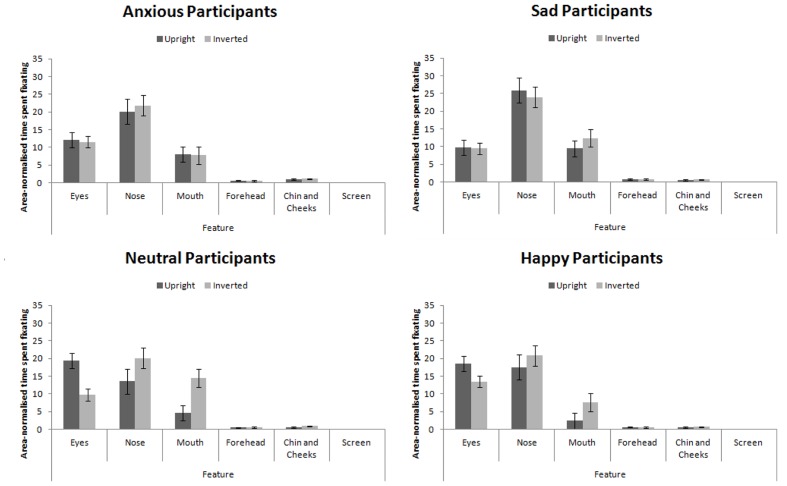
**Area-normalized time spent fixating in each AOI split by facial orientation and mood of participant**.

Proportion of time spent fixating in each AOI collapsed across expression (as expression did not interact with participant mood, see below) is presented in **Figure 5**. We analyzed the data using a 4 × 4 × 2 × 6 mixed-subjects ANOVA with the between-subjects factor of participant mood and within-subjects factors of facial expression, facial orientation, and feature. Here we present results relating to our hypotheses for clarity. For the full results, please contact the correspondence author.

This analysis revealed a main effect of feature, *F*(5,280) = 123.75, *MSE* = 270.00, *p* < 0.001, $\eta_{p}^{2}$ = 0.69 which revealed the standard hierarchy of features: the eyes and nose were fixated upon more than the mouth, forehead, chin and cheeks (all *p*s < 0.001). This effect interacted with participant mood, *F*(15,280) = 2.25, *MSE* = 270.00, *p* = 0.042, $\eta_{p}^{2}$ = 0.11. Šidák-corrected pairwise comparisons^4^ revealed that sad and anxious participants viewed the nose more than the eyes (*p*s < 0.01) whereas this difference was not significant for happy or neutral participants (*p*s > 0.98). Sad and anxious participants viewed the eyes less than happy and neutral participants (*p*s) < 0.01).

We also replicated the finding that feature viewed interacts with facial orientation (Barton et al., 2006; Hills et al., 2013), *F*(5,280) = 5.79, *MSE* = 161.28, *p* = 0.004, $\eta_{p}^{2}$ = 0.09: In inverted faces, the nose received greater fixation than the eyes (*p* < 0.001), but there was no difference in fixation to these features when viewing upright faces (*p* > 0.52).

#### Recognition Accuracy

The recognition accuracy measure, *d′*, was calculated using the Macmillan and Creelman (2005) method. *d′* combines hit rate and false alarm rate according to the formula:

$$d^{\prime }=z\left(Hitrate\right)-z\left(Falsealarmate\right)$$

**FIGURE 6:**
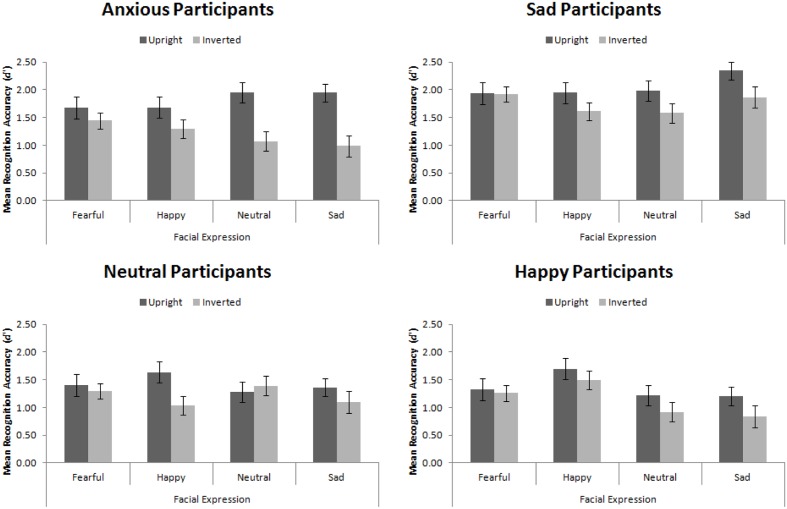
**Mean recognition accuracy for happy, sad, fearful, and neutral upright and inverted faces split by participant mood**. Error bars represent standard error of the mean.

For this Experiment, *d′* ranges from 0 to 3.67, whereby 0 is recognition at chance levels and 3.67 is recognition performance with zero errors^5^. The trend of these data, summarized in **Figure 6**, replicates that of Hills et al. (2011).

Replicating Hills et al. (2011), we found a main effect of participant mood, *F*(3,56) = 5.25, *MSE* = 1.98, *p* = 0.003, $\eta_{p}^{2}$ = 0.22. Šidák-corrected pairwise comparisons demonstrated that sad participants were significantly more accurate than happy (*p* = 0.004) and neutral (*p* = 0.012) participants, but not than anxious participants (*p* = 0.193). This main effect interacted with facial expression, *F*(9,168) = 3.83, *MSE* = 0.22, *p* < 0.001, $\eta_{p}^{2}$ = 0.17. While no simple effects were significant (all *p*s > 0.05), the pattern suggested that sad, neutral, and anxious participants recognized all faces equally, whereas happy participants had an advantage for recognizing happy faces.

While we found the standard face-inversion effect, in which upright faces (*M* = 1.66, *SE* = 0.08) were recognized more accurately than inverted faces (*M* = 1.32, *SE* = 0.07), *F*(1,56) = 27.14, *MSE* = 0.52, *p* < 0001, $\eta_{p}^{2}$ = 0.33, we did not find that mood interacted with facial orientation, *F*(3,56) = 1.98, *MSE* = 0.52, *p* = 0.127, $\eta_{p}^{2}$ = 0.10, replicating Experiment 1. This indicates that mood did not affect the amount of configural encoding engaged in. However, facial orientation did interact with expression, *F*(3,168) = 4.35, *MSE* = 0.20, *p* = 0.006, $\eta_{p}^{2}$ = 0.07. When viewing fearful faces, there was no FIE (*p* > 0.98), however, the FIE was significant when viewing all other types of faces (*p*s < 0.05). This final result indicates the primal importance of fearful expressions. The facial recognition system is finely tuned for rapid detection of threatening stimuli (Mogg and Bradley, 1998, 2002) and fearful expressions represent threat (though not of the stimuli itself but of something else nearby). The expression is basic enough that it is processed accurately and quickly using expert and non-expert processing.

Taken together, the results from Experiment 3 indicate that sad participants are more accurate at face recognition than happy participants, consistent with Experiment 1. They also show an eye-movement pattern in which they tend to avoid looking at the eyes, spending more time viewing the nose and mouth than happy participants do. The consequence of avoiding looking at the eyes leads sad participants to process other facial features more deeply. These results are consistent with Experiment 2. While the anxious participants also avoid looking at the eyes, we did not find convincing evidence that they were scanning other features. This, therefore, indicates a difference between sadness and anxiety: sadness involves processing other facial features, whereas anxiety simply involves avoiding the eyes. This extra processing in sad participants thereotically leads to greater recognition accuracy.

## Experiment 4

The results presented thus far indicate that sad and dysphoric participants are more accurate at face recognition tasks than happy and neutral participants. The results are consistent with a pattern of facial sampling that suggests sad participants are viewing more features of a face than happy participants do. Certain features are more diagnostic in the recognition of faces than others (e.g., Vinette et al., 2004). However, the most diagnostic features for faces of different races are not the same (Ellis et al., 1975). If sad participants look at more features of a face than happy participants, it is highly likely that they will sample features that are more diagnostic in the recognition of other-ethnicity faces (see e.g., Hills and Lewis, 2006). This would lead them to be less susceptible to the own-ethnicity bias. Johnson and Fredrickson (2005) have found that happy moods reduced the own-ethnicity bias relative to neutral and fearful participants. We, therefore, anticipate that both sad and happy participants would show a reduced own-ethnicity bias, but that sad participants would be more accurate overall at face recognition than happy participants.

### Method

#### Participants and Materials

Sixty (40 female) ethnically White undergraduate students from Anglia Ruskin University aged between 18 and 50 years of age participated in this experiment as a partial fulfillment of a course requirement. All participants self-reported that they had normal or corrected vision. We used 40 (20 ethnically Black or Asian)^6^ face identities from the NimStim database used in Experiment 3. All stimuli were presented in the same way as in Experiment 3 and the same eye-tracking equipment was used.

#### Design and Procedure

We employed a 4 × 4 × 2 mixed-subjects design with the between-subjects factor of participant mood (happy, sad, anxious, and neutral) and the within-subjects factors of facial expression (happy, sad, fearful, and neutral) and facial ethnicity (own- and other-ethnicity). All other aspects of the design and procedure were the same as in Experiment 3. This study received full ethical approval from the Science and Technology Research Ethics Panel at Anglia Ruskin University.

### Results and Discussion

The data treatment and presentation followed the same structure as that of Experiment 3. The mood induction was successful, *F*(3,56) = 31.82, *MSE* = 31.99, *p* < 0.001. Bonferroni-corrected *post hoc* tests demonstrated that happy induced participants were happier than all other participants (all *p*s < 0.001) and that sad and anxious induced participants were sadder than neutral and happy participants (all *p*s < 0.001). Numerically, sad induced participants were sadder than anxious induced participants (*p* = 0.154). This demonstrates that our mood induction was effective.

#### Eye-Tracking

**FIGURE 7:**
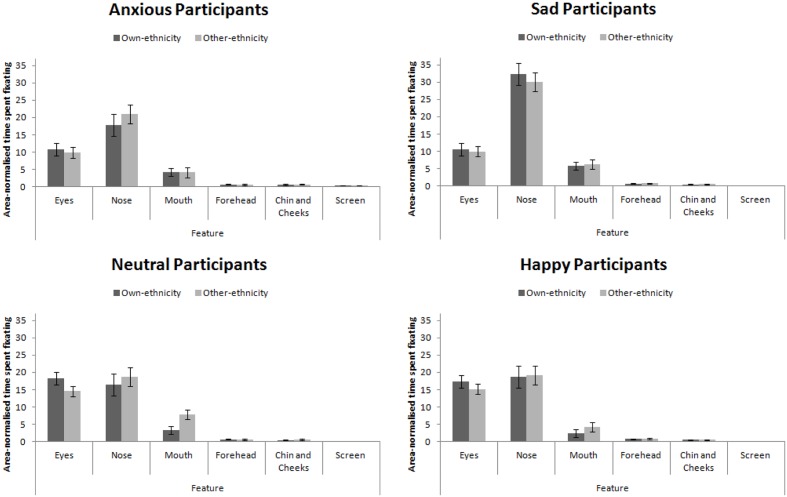
**Area-normalized time spent fixating in each AOI split by face ethnicity and mood of participant**.

The eye-tracking analysis was performed in a similar manner to Experiment 3. Mean proportion of time spent fixating in each AOI is presented in **Figure 7**. This figure collapsed across expression (as expression did not interact with participant mood, see below). A 4 × 4 × 2 × 6 mixed-subjects ANOVA with the between-subjects factor of participant mood and within-subjects factors of facial expression, facial ethnicity, and feature. This analysis revealed a main effect of feature, *F*(5,280) = 180.00, *MSE* = 679.79, *p* < 0.001, $\eta_{p}^{2}$ = 0.76 that revealed the standard hierarchy of features: the eyes and nose were fixated upon more than the mouth, forehead, chin and cheeks (all *p*s < 0.001). This effect interacted with participant mood, *F*(15,280) = 5.57, *MSE* = 679.79, *p* < 0.001, $\eta_{p}^{2}$ = 0.23. Šidák-corrected pairwise comparisons revealed that sad and anxious participants viewed the nose more than the eyes (*p*s < 0.01) whereas this difference was not significant for happy or neutral participants (*p*s > 0.91). Sad and anxious participants viewed the eyes less than happy and neutral participants (*p*s) < 0.01). Sad participants viewed the nose more anxious, happy, and neutral participants (all *p*s < 0.001). Anxious participants viewed the screen more than sad, happy, and neutral participants (all *p*s < 0.05). Facial ethnicity did not interact with feature, *F*(15,280) = 2.00, *MSE* = 258.47, *p* = 0.147, $\eta_{p}^{2}$ = 0.04.

#### Recognition Accuracy

**FIGURE 8:**
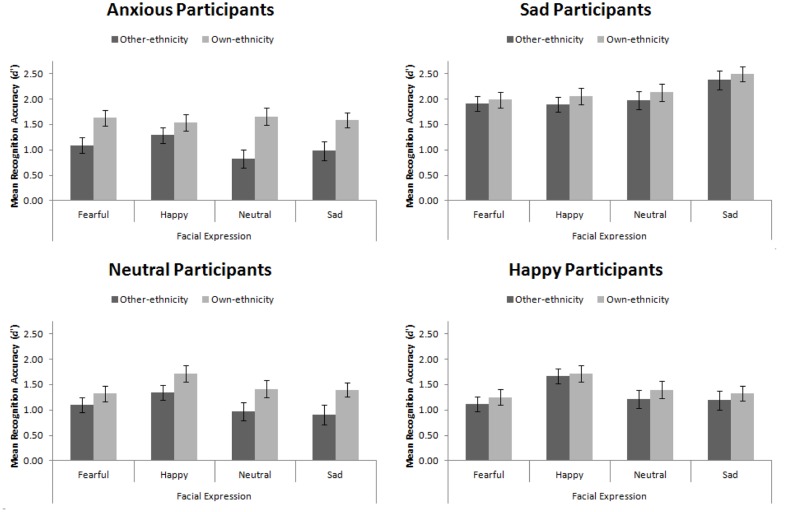
**Mean recognition accuracy for happy, sad, fearful, and neutral own- and other-ethnicity faces split by participant mood**. Error bars represent standard error of the mean.

Mean recognition accuracy, *d′*, for Experiment 4 is presented in **Figure 8**. Replicating Experiment 3, we found a main effect of participant mood, *F*(3,56) = 16.08, *MSE* = 1.16, *p* < 0.001, $\eta_{p}^{2}$ = 0.46. Šidák-corrected pairwise comparisons revealed that sad participants were significantly more accurate than happy, neutral, and anxious participants (all *p*s < 0.001). This main effect interacted with facial expression, *F*(9,168) = 3.94, *MSE* = 0.25, *p* < 0.001, $\eta_{p}^{2}$ = 0.17. While no simple effects were significant, the pattern suggested that sad, neutral, and anxious participants recognized all faces equally, whereas happy participants had an advantage for recognizing happy faces.

We found the standard own-ethnicity bias, in which own-ethnicity faces (*M* = 1.66, *SE* = 0.06) were recognized more accurately than other-ethnicity faces (*M* = 1.37, *SE* = 0.06), *F*(1,56) = 22.75, *MSE* = 0.46, *p* < 0.001, $\eta_{p}^{2}$ = 0.29. As hypothesized, we found that mood interacted with ethnicity of the face, *F*(3,56) = 2.86, *MSE* = 0.46, *p* = 0.045, $\eta_{p}^{2}$ = 0.13. Šidák-corrected pairwise comparisons were used to explore this interaction. We found a significant own-ethnicity bias in our neutral, *t*(14) = 2.91, *p* = 0.011, and anxious participants, *t*(14) = 4.71, *p* < 0.001, but not in our happy participants, *t*(14) = 0.83, *p* = 0.42, consistent with Johnson and Fredrickson (2005), nor our sad participants, *t*(14) = 1.34, *p* = 0.202, consistent with our hypothesis.

The results from Experiment 4 confirm the findings from Experiment 3: sad participants sample more of a face than happy participants. Due to sad participants sampling more of the face, they sample facial features that are diagnostic in the recognition of other-ethnicity faces. This means that they are able to code faces of other-ethnicities in a manner that is more suitable for those faces and therefore reduces the magnitude of their own-ethnicity bias. While the anxious participants in this experiment viewed the eyes less than the happy and neutral participants, they did not view other features more so than those participants, consistent with Experiment 3. In fact, they viewed the screen (rather than the face) more than other participants, indicating an avoidant strategy in this task.

In this experiment, we found that both sad and happy participants showed a reduced own-ethnicity bias. The reasons for the reduced own-ethnicity bias is likely to be different in both groups given that there were no appreciable eye movement differences between the happy and neutral participants. Potentially we did not have sufficient power to find that happy participants would be more accurate than the neutral participants in recognizing other-ethnicity faces. Nevertheless, we hypothesize that these two effects are based on different mechanisms. Johnson and Fredrickson (2005) indicate that happy participants have a more inclusive thought process (Fredrickson, 2001). This effect, these authors assume, leads to enhanced holistic processing which improves the recognition of other-ethnicity faces (but has no effect on the recognition of own-ethnicity faces according to them). We believe that the effect reported by Johnson and Fredrickson (2005) is related to the fact that happy participants used more inclusive social categorisation strategies (Isen et al., 1992; Dovidio et al., 1995). By having a more inclusive in-group, other-ethnicity faces may be attended to more and this leads to enhanced accuracy (Hugenberg et al., 2007). We believe that what drives sad participants to show a reduced own-ethnicity bias is a differential scanning strategy whilst viewing faces as borne out by our data and the fact that sad participants do not show enhanced configural (Experiment 1) nor holistic (Curby et al., 2012) processing.

## General Discussion

We aimed to explore why sad people show a memory advantage for faces (Hills et al., 2011). In Experiment 1, we established that dysphoria was correlated with face recognition accuracy and defocused attention. However, defocused attention was not correlated with face recognition accuracy. Dysphoria was not correlated with effort nor configural processing. From this, we assumed that mood affected face perception through an alternative attentional mechanism that we hadn’t tested. Therefore, we explored whether mood affected which features were used when processing faces. Using a paradigm borrowed from Hills and Lewis (2011b), we replicated the finding that sad participant’s detected changes made to the head shape and the nose more accurately than control participants but did not detect changes made to the eyes as well. We extended the original finding by demonstrating a highly similar pattern in anxious participants. Furthermore, we demonstrated that sad, anxious, and control participants used the mouth an equal amount. Taken together, these findings suggest that sad and anxious participants might be showing more defocused attention and looking at different parts of the face, but they are still showing a feature hierarchy in which the mouth is not diagnostic to recognition.

In Experiment 3, we used eye-tracking during a face recognition procedure in order to verify that mood affected the way in which features were sampled. Our results clearly show that participants induced into sad or anxious moods did not look at the eyes as much as happy or control participants. Mood did not interact with facial orientation. Taken together, these results indicate that sad mood is not related to the amount of configural processing engaged in. Rather, mood is related to a pattern of eye movements over a face in which features other than the eyes are processed more deeply. We assumed that this would cause participants to sample parts of the face that are more diagnostic in the recognition of other-ethnicity faces. This logic is derived from the fact that certain facial features are more diagnostic in certain categories of faces: eye color is diagnostic for White faces, whereas the nose is relatively more diagnostic for Black faces (Ellis et al., 1975; Shepherd and Deregowski, 1981). If sad participants use features other than the eyes for recognition, then they may show a reduced own-ethnicity bias. In Experiment 4, we found precisely this pattern.

All together, these results are entirely consistent with a model of mood affecting face recognition through altering the way faces are scanned and what information is extracted. This may be a result of defocused attention, or more plausibly, a result of the desire not to make eye contact, while remaining motivated to be accurate (given that there is substantial evidence for sad participants to be motivated to process information deeply, Bless et al., 1990; Bodenhausen, 1993; Martin et al., 1993). Anxious participants do not have this motivation to be accurate and therefore avoid looking at the diagnostic features. If the faces are scanned in an appropriate manner, sad participants will demonstrate increased recognition accuracy relative to happy participants. Crucially, this is a result of participants engaging with the faces during the learning task, because we have previously demonstrated that this pattern of recognition occurs when the learning is incidental rather than intentional. When the learning is intentional, mood does not affect recognition performance (Jermann et al., 2008; see also Hills et al., 2011, Experiment 3) or produces the opposite effects (Xie and Zhang, 2015). In intentional learning, all participants have enhanced motivation to process faces accurately: sad participants’ enhanced motivation to be accurate does not enhance their accuracy above that of participants in other moods. Therefore, the effect of sad mood enhancing face recognition accuracy will only occur under incidental learning conditions as the mood is leading to enhanced motivation rather than the task constraints.

While we have consistently found the present set of results and can explain these results in a simple framework based around information input, some of the findings we have reported here are inconsistent with findings from other authors. Xie and Zhang (2015) reported that mood was associated with holistic processing measured by the composite face effect. Scores on the composite face task do not correlate strongly with measures of configural processing such as the face-inversion effect (Herzmann et al., 2010). While there are no consistently agreed definitions of holistic and configural processing, three components of configural processing have been identified (Maurer et al., 2002). The first is processing the overall configuration of a face. This part of configural processing is necessary to engage the subsequent expert face processing mechanisms. Second-order relational information processing and holistic processing can be engaged when the first-order configural matches a face. Holistic processing, in which faces are processed as a whole (Burton et al., 2015), is likely to be the expert face processing mechanism. Inversion disrupts all three parts of configural processing, whereas the composite face effect only measures holistic processing. Therefore, the lack of consistency between Xie and Zhang’s (2015) findings and ours is not a surprise. Furthermore, the amount of holistic processing engaged in does not typically correlate with face recognition accuracy (Konar et al., 2010), therefore we would not expect to find the strong correlations found by Xie and Zhang (2015). A final reason for the difference refers to the difference in testing: implicit memory is affected by mood in the way we have described but explicit memory is not (Hills et al., 2011).

Our results have an implication in the way that the effects of mood are considered. While many researchers consider the effect of mood to be a social and interpersonal one, we have demonstrated that mood affects eye movements and therefore the input of information into the cognitive system. This therefore means that mood may affect the entire cognitive system and potentially some of the higher level effects of mood may actually be the result of low level perceptual systems.

In sum, we have found that mood affects eye movements when looking at faces. Sad mood and anxiety cause participants to avoid looking at the eyes. This causes sad participants to sample other features. Because sad participants appear to show an adaptive response where they attempt to repair their mood by being accurate (Bless et al., 1992; Bodenhausen, 1993; Martin et al., 1993), they code faces more deeply than is typical, by sampling additional features, and this leads to improved face recognition abilities. Therefore, the memory advantage for faces that sad people show is due to an unusual allocation of attention.

## Ethics Statement

This study was carried out in accordance with the recommendations of the British Psychological Society with written informed consent from all subjects. All subjects gave written informed consent in accordance with the Declaration of Helsinki. The protocol was approved by the Faculty of Science and Technology’s Research Ethics Panel at Bournemouth University (Experiments 1 and 2) and the Faculty of Science and Technology’s Research Ethics Panel at Anglia Ruskin University (Experiments 3 and 4).

## Author Contributions

PH: supervisor for all three students. Wrote the paper, re-analyzed the data. ZM: designed and drafted Experiment 1. IY: designed, collected all data, and drafted Experiment 2. IG: assistant in design, collected all data, and drafted Experiment 3.

## Conflict of Interest Statement

The authors declare that the research was conducted in the absence of any commercial or financial relationships that could be construed as a potential conflict of interest.

## Footnotes

1 The terms configural and holistic processing have been used interchangeably by researchers, yet there is evidence that they refer to different processes, given substantial evidence indicates that expert face processing does not rely on fine-grained configural information (Hole et al., 2002; Mondloch and Desjarlais, 2010; for a review see Burton et al., 2015). Indeed, tests of configural processing do not correlate with other tests of holistic processing strongly (see e.g., Wilhelm et al., 2010). This is likely due to the lack of consistent definitions of these concepts (Burton et al., 2015) and the subtle differences in these concepts. For the purpose of this article, we use the term “configural processing” to refer to the three types of processing identified by Maurer et al. (2002).

2 Of course, it could be that sadness simply slows processing down and therefore longer reaction time may not be a sign of motivation but rather slow processing.

3 Note that this check does not check whether the anxious mood induction procedure was effective. However, given the mood induction procedure was effective for sadness, we have no reason to assume it wouldn’t be for anxiety. Similarly, there were differential effects (see the results) for sad induced and anxious induced participants indicating that the procedure was effective (despite there being some similar effects for both sad and anxious induced participants).

4 The Šidák correction were more appropriate for this analysis than the Bonferroni correction because the number of comparisons makes the Bonferroni correction prohibitively conservative and unnecessarily increases the risk of a Type II Error (see Field, 2010).

5 *d*′ can range to infinity in experiments with more stimuli, but for the number of stimuli used in the present experiment, 3.67 was the maximum value.

6 In preliminary analyses, we did not find any differences in any dependent measures across Black and Asian faces, therefore these were combined to form an other-ethnicity group for our White participants.
